# Genome survey and genetic characterization of *Acacia pachyceras* O. Schwartz

**DOI:** 10.3389/fpls.2023.1062401

**Published:** 2023-02-16

**Authors:** Nazima Habibi, Fadila Al Salameen, Nishant Vyas, Muhammad Rahman, Vinod Kumar, Anisha Shajan, Farhana Zakir, Nasreem Abdul Razzack, Bashayer Al Doaij

**Affiliations:** ^1^ Environment and Life Science Research Centre, Kuwait Institute for Scientific Research, Kuwait, Kuwait; ^2^ Department of Immunology, Logical Life Sciences, Pune, India

**Keywords:** whole genome sequencing, gene annotation, woody tree, microsatellite, SSR primers, transposable element, flow cytometry

## Abstract

*Acacia pachyceras* O. Schwartz (Leguminoseae), a woody tree growing in Kuwait is critically endangered. High throughput genomic research is immediately needed to formulate effective conservation strategies for its rehabilitation. We therefore, performed a genome survey analysis of the species. Whole genome sequencing generated ~97 Gb of raw reads (92x coverage) with a per base quality score above Q30. The k-mer analysis (17 mer) revealed its genome to be 720Mb in size with an average guanine-cytosine (GC) ratio of 35%. The assembled genome was analyzed for repeat regions (45.4%-interspersed repeats; 9%-retroelements; 2%-DNA transposons). BUSCO assessment of completeness of genome identified 93% of assembly to be complete. Gene alignments in BRAKER2 yielded 34,374 transcripts corresponding to 33,650 genes. Average length of coding sequences and protein sequences were recorded as 1,027nts and 342aa, respectively. GMATA software filtered a total of 901,755 simple sequence repeats (SSRs) regions against which 11,181 unique primers were designed. A subset of 110 SSR primers were PCR validated and demonstrated for its application in genetic diversity analysis of *Acacia*. The SSR primers successfully amplified *A. gerrardii* seedlings DNA depicting cross transferability among species. The principal coordinate analysis and the split decomposition tree (bootstrapping runs of 1000 replicates) distributed the *Acacia* genotypes into two clusters. The flow cytometry analysis revealed the *A. pachyceras* genome to be polyploid (6x). The DNA content was predicted as 2.46 pg, 1.23 pg, and 0.41 pg corresponding to 2C DNA, 1C DNA and 1Cx DNA, respectively. The results provide a base for further high throughput genomic studies and molecular breeding for its conservation.

## Introduction

Loss of plant diversity due to habitat loss, fragmentation, and degradation, overexploitation, pollution, and anthropogenic climate change is infusing worldwide concern (Corlett, 2016). The harsh climatic conditions further intensify the impact in arid lands including the Arabian Peninsula (Al Salameen et al., 2018; Mosa et al., 2019; Al Salameen et al., 2020; Habibi et al., 2020; Habibi et al., 2022a). Kuwait offers a valuable genetic pool of native flora encompassing genes related to salt and stress tolerance, however the plant diversity faces serious consequences of degradation (Omar et al., 2001; Asem and Roy, 2010; Al Salameen et al., 2018b; Al Salameen et al., 2020; Al Salameen et al., 2022). Efforts have been initiated in Kuwait to restore its native vegetation, the country is a signatory of Convention on Biological Diversity (CBD), and therefore it’s one of its mandate to genetically characterize the declining species for effective conservation management (CBD, 1992).

The first critical step in protecting and managing threatened species is to investigate their ecological and evolutionary characteristics (Al Salameen et al., 2018b; Habibi, 2019; Habibi et al., 2020). Genomic tools have emerged as an valuable tool in all aspects of conservation genetics (Byrne, 2018). Next generation sequencing (NGS) is a powerful method that answers the fundamental evolutionary biology questions, however its application at a wider scale is hampered due to the still high cost of whole genome sequencing and the demand for heavy computational resources. Further, the scarce genomic resources of non-model species limits its implementation to a variety of plant species (Fuentes-Pardo and Ruzzante, 2017). The NGS based genome survey or sequencing at a lower depth is an imperative and cost-effective approach to obtain genetic information of the desired organism without prior knowledge of its sequence (Kirkness et al., 2003). The preliminary genomic characteristics gained through this method is useful to generate a species-specific resource as well as elements for comparative genomic analysis (Straub et al., 2012). The baseline information obtained by genome skimming sets a foundation for large scale, high throughput sequencing, species identification, hybridization, kinship and evolutionary history analysis (Fuentes-Pardo and Ruzzante, 2017).

*Acacia pachyceras* Schwartz (Leguminosae) is an endemic species native to Kuwait. There is only one surviving specimen in the region (Suleiman, 2017). A desert dweller, highly stress tolerant with the capability of nitrogen fixation is a highly desirable candidate for desert rehabilitation (Nevill et al., 2013; Suleiman, 2017; Gugger et al., 2018; Blyth et al., 2020). Apart from this the species have been reported to bear medicinal properties (El-Wahab et al., 2008). In Kuwait, it has been designated as “Near to threatened” by the World Conservation Monitoring Centre (Oldfield et al., 1998). It is therefore crucially important to formulate conservation strategies for this tree species (Habibi et al., 2020). Efforts for its restoration have been initiated (Nevill et al., 2013; Suleiman, 2017; Suleiman et al., 2018; Suleiman et al., 2019), however genomic information are completely lacking. We therefore performed the genome survey analysis to predict its genome size, perform gene annotations and filter repetitive sequences within the genome. Primers flanking the simple sequence repeats (SSR) motifs were also designed and validated for genetic diversity analysis.

## Materials and methods

### Sampling and DNA isolation

**Figure 1:**
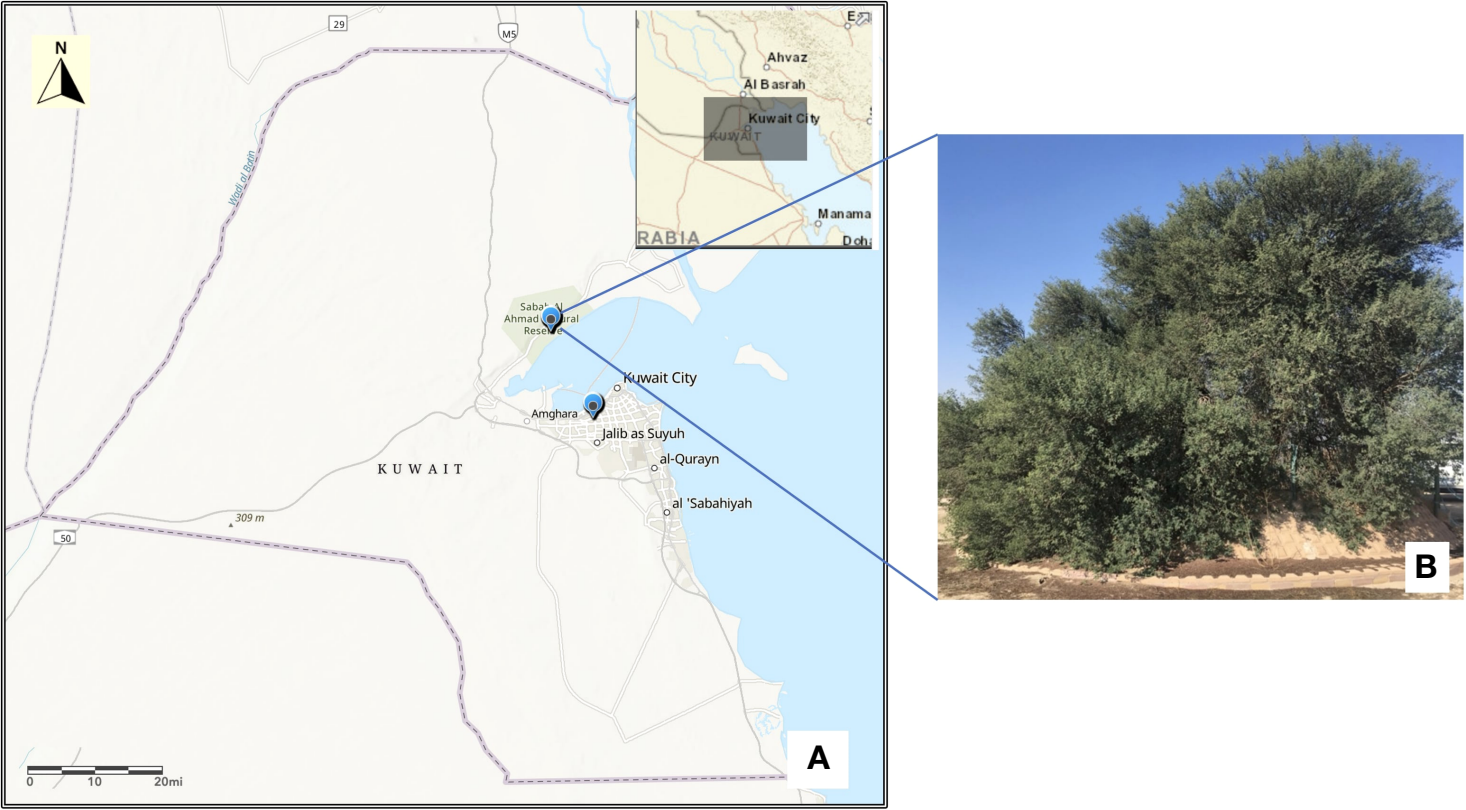
Map of Kuwait **(A)** showing the location of Acacia tree in Sabah Al-Nature Reserve and KISR greenhouse. Map was drawn using the ArcGIS v10.4.1. **(B)** The single mature tree of *Acacia pachyceras* growing in the Sabah Al-Nature Reserve area of Kuwait.

For the present investigation, young leaf samples from the only persisting mature tree specimen of *Acacia pachyceras* protected in the Sabah Al Nature Reserve (SANR) of Kuwait (**Figure 1**) was collected during January 2020. Three to four seed pods from this tree were also gathered, their embryo excised and germinated at KISR laboratories, out of which only one survived. Young leaves were snipped from the tip of this one year old seedling for DNA isolation. Leaf samples from morphologically distinct *Acacia* genotypes growing in close vicinity of the mother tree (north west, n=5; and north east, n=5) in the SANR were also collected for genetic diversity analysis. Approximately 2-3 months old seedlings of *Acacia gerrardii* (Suleiman, 2017; Suleiman et al., 2018) growing (n=5) under the green house of Kuwait Institute for Scientific Research (KISR) were also sampled for comparison with field grown accessions. GPS coordinates of all the samples were recorded (**Table S1**). In total 17 samples were collected and DNA was isolated from the leaf samples as per the CTAB protocol described elsewhere (Habibi et al., 2022b). DNA quality, purity and quantity were estimated through spectrophotometric (Nanodrop UV/Vis spectrophotometer, ThermoFisher) and fluorometric (Qubit, Invitrogen, WA) methods as per the manufacturer’s instructions.

### Ploidy and genome size by flow cytometry

Plant tissues from the standard *Panicum miliaceum* (2n=4x=36; 2C=2.09 pg DNA) (Kubešová et al., 2010) *A. pachyceras*, and *A. gerrardii* were chopped into small pieces ranging between 0.3 to 1.0 mm into a Petri dish filled with a mix of OttoI (0.1 M citric acid + 0.50% Tween20) and OttoII (0.40M of Na_2_HPO_4_.12H_2_O) (1:1). Whole mixture was filtered through 42-μm nylon mesh and centrifuged at 5000 rpm and incubated at 40°C. The pellet was collected and resuspended in OttoI. The suspensions were subjected to RNA degradation using RNase (Sigma Aldrich) for 30 minutes. Nuclear DNA was stained by adding 75 µl of propidium iodide (Sigma Aldrich). Flowcytometry based analysis of stained nuclei was performed on 3 laser flow cytometer MACSquant10 analyzer (Miltenyi Biotech GMBH). A total of 100,000 events were recorded for each sample and florescence was measured in B2 channel and median florescent intensities were measured. Final data was normalized and analyzed by FlowJo™ v10.8 Software (BD Life Sciences). The absolute DNA amount of a sample was calculated based on average primary peak value as per the method defined by (Doležel and Bartoš, 2005).

### Genome sequencing and K-mer analysis

The single DNA sample from the mature tree of *A. pachyceras* was lyophilized and shipped to the Beijing Genomics Institute (BGI), Hong kong for paired-end sequencing. Post-quality checks at BGI, the DNA was tagmented and subjected to library preparation. DNA fragments were end repaired, 3’A tailed and ligated with Illumina adapters (Khan et al., 2016). Thereafter the libraries were purified through Agencourt AMPure XP magnetic beads (Beckman Coulter Genomics, Brea, CA, USA) (Habibi et al., 2021). Sequencing was performed on the Illumina HiSeq 2500 (Illumina, San Diego, US) platform using the 2 x 250 cycle chemistry. Quality parameters for the raw data were accessed through FASTQC version 0.119 (Andrews, 2010). The raw reads were trimmed using the Trimmomatic v. 0.17 (Bolger et al., 2014). The filtered high-quality sequences were initially assembled by Platanus-allee 2.0 (Kajitani et al., 2019). JELLYFISH 2.1.4 was used to conduct the K-mer analysis (Marçais and Kingsford, 2011). Genome size estimation was done based on k-mer frequency distribution and the number of 17-mer.

### *De novo* assembly and gene annotation

For gene annotation the high-quality paired-end DNA sequencing data was used for *de-novo* assembly of *Acacia pachyceras* genome using MaSuRCA-4.0.3 (Zimin and Salzberg, 2022). The primary assembly was filtered to remove scaffolds shorter than 500 bp. A *de-novo* repeat library for filtered assembly was constructed using RepeatModeler2.0.3 (Flynn et al., 2020) that included RECON (Bao and Eddy, 2002), RepeatScout (Price et al., 2005), and Tandem Repeat Finder (Benson, 1999). The repeat library was then subjected to RepeatMasker 4.1.3 to find and mask the repeats in the assembled genome using rmblast as the default search engine (Smit and Hubley, 2008). The BRAKER2 pipeline was used to perform gene prediction by integrating *ab initio* gene prediction, based on plant protein sequences, combining both GeneMark-EP and AUGUSTUS 3.2.0 (Brůna et al., 2021). The plant protein sequences from various species used for gene prediction were downloaded from the OrthoDB v10 database. The ProtHint 2.5.0 protein mapping pipeline was used for generating required hints from the plant protein sequences for BRAKER (Hoff et al., 2018). The assembled scaffolds and generated hints from the protein sequences were used for obtaining initial gene structures using the GeneMark-ET tool. The initial gene structures were then used for training by AUGUSTUS to produce the final gene predictions. The predicted genes were filtered to retain those with fully supported hints. GC content of the genome sequence was estimated (Gurevich et al., 2013). The BUSCO v3.0.2 (Benchmarking Universal Single-Copy Orthologs) tool was used to evaluate the completeness of the genome (Simão et al., 2015).

### Identification of microsatellite motifs and genetic diversity analysis

The GMATA 2.0 tool was used to filter microsatellite regions in the genome (Wang and Wang, 2016). The recognition criteria for the number of di-, tri-, tetra-, penta- and hexa-nucleotides repeats were set as 5 and above. The in-built Primer 3 (v 3.0) in GMATA was used to design primers against the filtered SSR motifs (Untergasser et al., 2012). The parameter setting of primer design was 18 ~ 23 bp primer size, annealing temperature at 57–62 °C, GC content at 30–70% and 100 ~ 400 bp final product length. A total of 110 primers were randomly chosen and synthesized for PCR amplification (Thermo Fisher Scientific, Waltham, MA, USA). All the primers were dissolved in nuclease free water and adjusted to 1 μM working stock. PCR was conducted on 17 genotypes of *Acacia.* A reaction volume of 20μL was assembled in a 200 μL PCR plate comprising 10 ng of DNA, 1× PCR Master Mix (Solis BioDyne, Tartu, Estonia), and 0.3 μM of each forward and reverse primers. Thermal cycling was performed in a Veriti Thermal Cycler (Applied Biosystems, Grand Island, NY) by initially inactivating the DNA polymerase at 95°C for 12 mins, followed by 30 cycles of denaturation at 95°C for 30 sec, annealing at 55°C for 30 s and extension at 72°C for 50 sec (Mustafa et al., 2017). The PCR products were visualized on 2.0% agarose gel at 10 V/cm for 60 mins. Gel was visualized on a gel documentation system (Chemidoc MP, BioRad, USA) under UV light. All the samples amplified and produced clear, reproducible bands generating 186 loci, which were scored as present (1) or absent (0). A binary matrix (1/0) was generated and analyzed through the GenAlEx 6.5 software (Peakall and Smouse, 2006). Population structure was determined by the neighbor-net split decomposition network generated by SPLITS Tree 5.0 and bootstrapping runs of 1000 replicates (Kloepper and Huson, 2008) and principal coordinate analysis (PCoA).

## Results

This manuscript presents the genome sequence data of a rare and endangered tree species growing in Kuwait. The raw sequences were used to estimate the genome size, repeat content, gene numbers, GC % content and microsatellite primers distributed within the DNA of *Acacia pachyceras*. Primers designed against the SSR regions were used for genetic diversity analysis. Flow cytometric analysis was performed for ploidy identification and 2C based genome size prediction.

### Sequence filtering and k-mer analysis

**Figure 2:**
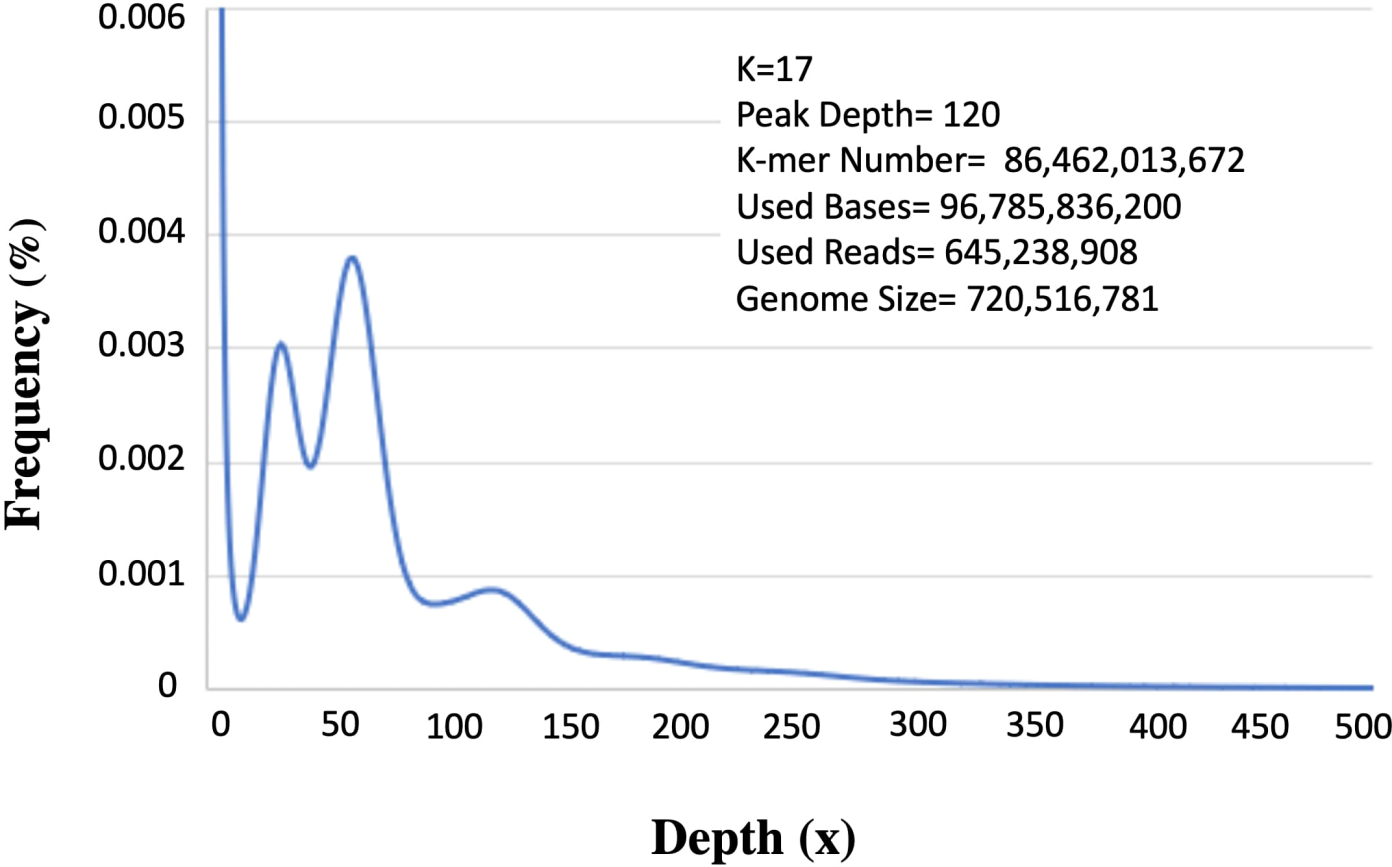
K-mer analysis for genome size prediction of *Acacia pachyceras.* The x-axis represents the depth and the corresponding frequencies are plotted on the y-axis. The values on the right-hand side panel shows the k-mer analysis parameters and the genome size (k-mer number/peak depth). Peaks at 25x and 50x are due to the repetitive and heterozygous genome sequences respectively.

Two 300-400 bp (insert size) paired-end libraries yielded 108 GB, raw reads. Per base quality score ranged between 34.0-37.0 (mean=36.0). Approximately 10% of low-quality data was filtered and the remaining 96 GB (ca. 89%) was used for downstream analysis. Among these about, 645 Mb qualified for further analysis. K-mer analysis provides an estimation of genome size based on substrings of length k contained within a biological sequence. Besides, this it also indicates low quality or contamination in the sequences. The 17-mer frequency analysis identified the main peak at 120 X depth based on the k-mer number of 86,462,013,672 (**Figure 2**). Upon dividing the k-mer number by 120 the genome size was predicted as ~720 Mb (720,516,781bp). The other peak at ½ of the main peak (50X) is most likely due to the high level of heterozygosity (6.84%), whereas an additional peak at a similar depth (25X) is because of repeat rates at the position of multiple integers (56.89%).

### *De-novo* assembly and gene prediction

**Figure 3:**
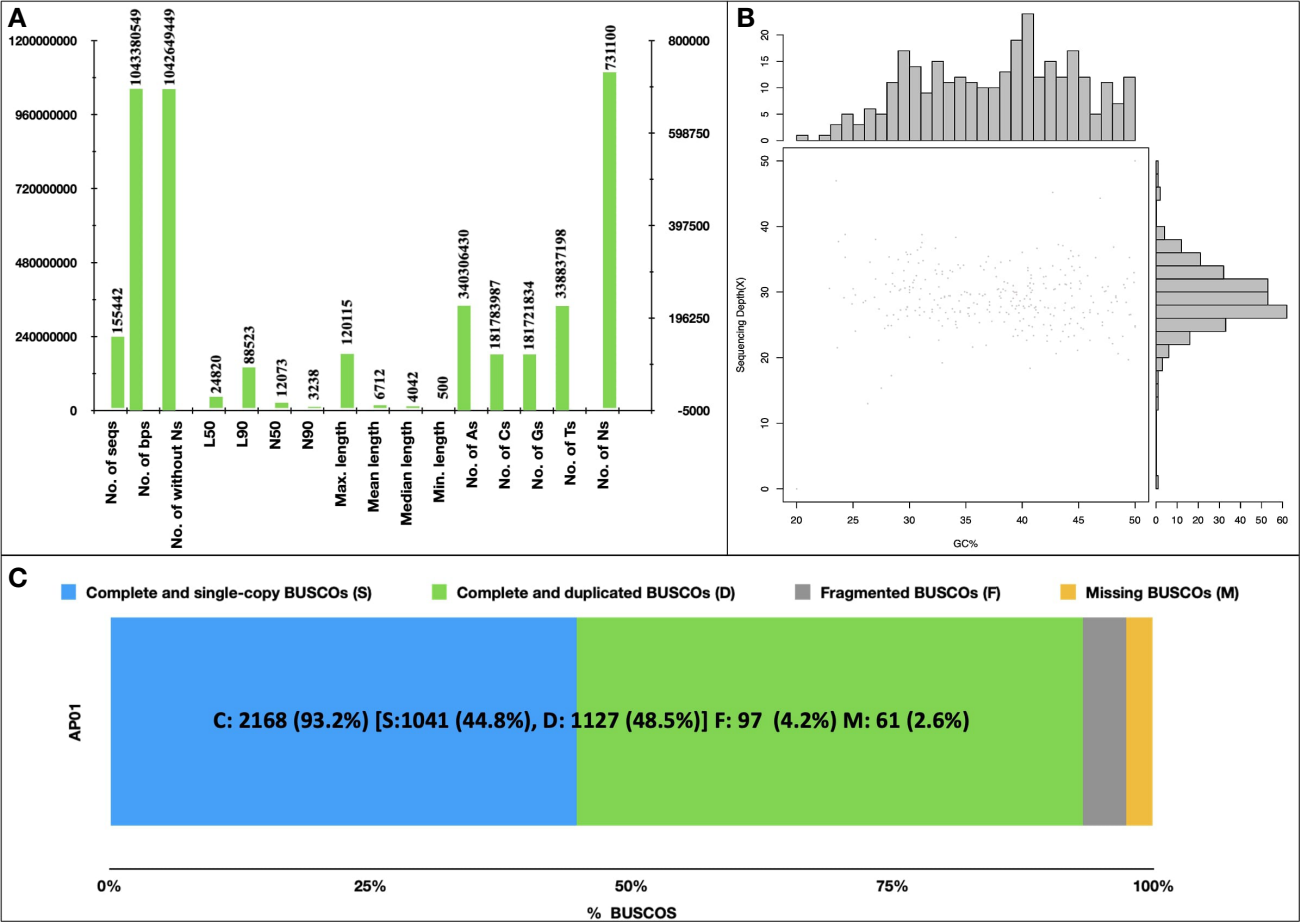
*De novo* Assembly of *Acacia pachyceras.* **(A)** Assembly statistics. Genome was assembled using MaSuRCA-4.0.3. **(B)** GC content of *Acacia pachyceras* genome. The x-axis represents the GC% and sequencing depth is plotted on y-axis. Sliding window at 10kb non-overlapping bases was used for GC% estimation. **(C)** BUSCO assessment of completeness of genome assembly. BUSCO was run in genome mode using metaeuk as the gene predictor. The x-axis presents the BUSCO % and the Y-axis denotes the genome name.

A total of 640 million paired-end reads were used to construct a raw genome assembly of *A. pachyceras*. The assembled genome consisted of 169,210 scaffolds (1.19 Gb). Removal of scaffolds < 500 Mb yielded a filtered assembly of ~1.043 Gb containing 155,442 scaffolds (**Figure 3A**). The number of sequences excluding Ns was 1,042,649,449 bp. The final assembly contained very low N content (~0.07%). The largest scaffold was 120,115 bp with an N50 of 12,073, N90 of 3,238, L50 of 24,820 and L90 of 88,523. The genome contained more adenosine bases (340,306,430) followed by thymine (338,837,198) > cytosine (181,783,987) and guanine (181,721,834) (**Figure 3A**). The GC content was predicted to be 34.84% employing a sliding window algorithm at every 10kb sequence. (**Figure 3B**). BUSCO assessment of completeness of genome revealed 93.2% of assembly to be complete (C: 2,168). There were 1,127 (48.5%) complete and duplicate BUSCOs (D), 1,041 (44.8%) complete and single copy BUSCOs (S), and a mere of 97 (4.2%) of fragmented BUSCOs (F). Total 2,368 BUSCOs were searched among which only 2.5% (67) were missed (M) (**Figure 3C**). The higher number of C as compared to F and M BUSCOs is suggestive of a high-quality genome assembly.

### Repetitive sequences, gene prediction and annotation

Generic repetitive DNA often assist in expression of typical coding sequences and important in organizing functions required for genome replication and progression to the following generation of cells. Therefore, we characterized the repetitive sequences in the assembled genome and found its total length as ~488.3 Mb, accounting for ~46.8% of the draft assembly. Among these, ~34% of the repeats were unclassified. DNA transposons corresponded to ~2%, whereas, retroelements corresponded to ~9% of the genome. A complete list of repeats along with the content in the draft genome has been shown in **Table 1**. Total interspersed repeats were 45.42%.

**Table 1:** Repeat annotation of the assembly.

Type of repeats	Number of elements	Length (bp)	% of sequence
Retroelements	152,396	93,651,204	8.98
SINEs:	0	0	0
Penelope	605	189,925	0.02
LINEs:	47,403	22,639,339	2.17
CRE/SLACS	0	0	0
L2/CR1/Rex	0	0	0
R1/LOA/Jockey	2,085	679,020	0.07
R2/R4/NeSL	0	0	0
RTE/Bov-B	11,643	2,305,609	0.22
L1/CIN4	32,808	19,409,845	1.86
LTR elements:	104,993	71,011,865	6.81
BEL/Pao	0	0	0
Ty1/Copia	56,208	38,366,290	3.68
Gypsy/DIRS1	40,831	29,349,199	2.81
Retroviral	4,347	821,852	0.08
DNA transposons	44,077	19,566,198	1.88
hobo-Activator	5,505	2,200,763	0.21
Tc1-IS630-Pogo	0	0	0
En-Spm	0	0	0
MuDR-IS905	0	0	0
PiggyBac	0	0	0
Tourist/Harbinger	5,330	1,440,189	0.14
Other (Mirage, P-element, Transib)	0	0	0
Unclassified	1,432,956	360,646,294	34.57
Total interspersed repeats		473,863,696	45.42
Rolling-circles	7,158	3,371,786	0.32
Small RNA	1,126	224,912	0.02
Satellites	0	0	0
Simple repeats	247,128	895,2077	0.86
Low complexity	40,734	1,954,051	0.19

Gene prediction using BRAKER2 pipeline based on *ab-initio* method, and ortholog protein sequence- resulted in a total of 123,610 genes corresponding to 127,367 transcripts. However, these include both supported and unsupported transcripts. The transcripts that are fully supported by hints are the most confident predictions. Hence, we used a script, ‘*selectSupportedSubsets.py*’ provided by the BRAKER2 pipeline to extract the most confident predictions from the complete list of transcripts. This resulted in a total of 34,374 transcripts corresponding to 33,650 genes. These set of transcripts were considered for further analysis. The statistics of most confident transcript and protein sequences are shown in **Table 2**. The mean length of the coding sequence was 1,027 nts (Max 11,262 nts; Min-201nts), whereas that of protein sequence was 342 aa (Max-3,754 aa; Min-67aa).

**Table 2:** Statistics of predicted transcript and protein sequences.

Parameters	Transcript	Protein
Total count	34,374	34,374
Total bp/aa	35,311,671	11,770,557
Minimum length	201	67
Maximum length	11,262	3,754
Mean length	1,027	342

### SSR mining, primer designing and genetic diversity analysis

**Figure 4:**
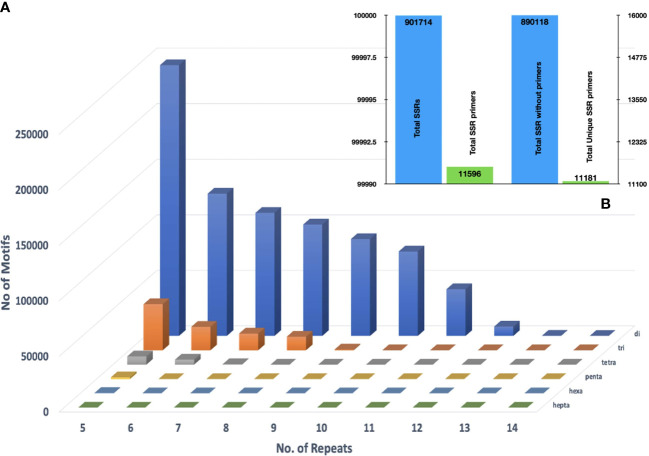
**(A)** Distributions of di- to hexanucleotide (SSR) motif types with repeat numbers ranging from 4 to 14 in the assembled genomic sequences of *Acacia pachyceras* **(B)** Primers designed against the filtered SSR motifs employing Primer 3.

Mining the assembled genome for microsatellite motifs in GMATA yielded 901,755 SSR motifs. Among these, dinucleotides were the largest in number (796,441; 88%), followed by the trinucleotides (90,769; 10.06%), tetranucleotides (12,435; 1.3%), pentanucleotides (1,651; 0.18%), and, hexanucleotides (418; 0.05%) (**Figure 4A**). In total 11,596 primer pairs were designed against these SSR motifs and 11,181 among them were unique (**Figure 4B**). Approximately 98.71% of SSR motifs were positioned in the genome that did not meet the standard primer designing parameters hence no primers could be designed for them. The GBS based SNP markers are recommended to be investigated to overcome this limitation.

**Figure 5:**
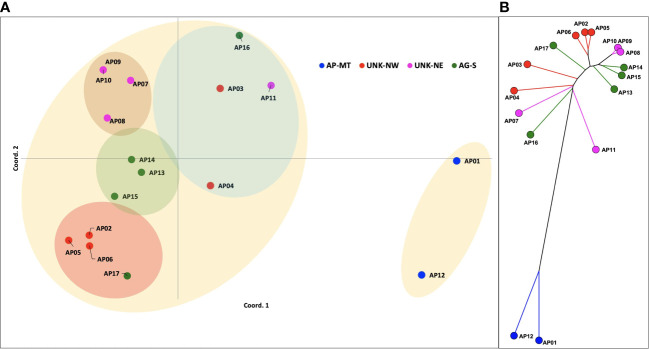
**(A)** Principal Coordinate Analysis (PCoA) plot of the pairwise genetic distances between 17 genotypes of *A. pachyceras* based on 189 polymorphic SSR loci. **(B)** A split decomposition network tree of seventeen Acacia genotypes. The splits tree v5 algorithm was used to construct the tree with 999 permutations and bootstrapping.

A subset of 110 primer pairs representing each repeat class were randomly chosen for PCR validation (**Table S2**). Amplification yielded 186 genetic loci for downstream analysis. Among, these 141 (75.65%) were polymorphic (PIC=0.27). These loci were imported in the GenAlEx v6.5 software for estimating the pairwise genetic distances, analysis of molecular variance (AMOVA) and principal coordinate analysis. The PCoA analysis revealed the *Acacia*accessions to be distributed into three clusters. Variations at 1^st^ 2^nd^ and 3^rd^ axis were 24.26%, 38.54% and 49.06%, respectively. The PCoA distributed the *Acacia* genotypes into a small and a large cluster (**Figure 5A**). The smaller cluster had only two genotypes AP01(single specimen of *A. pachyceras* growing in SANR) and AP12 (seedling of the mother tree). Rest all the genotypes formed a part of the larger cluster. The UNK-NE, UNK-NW and the AG-S existed as three overlapping sub-clusters within the larger cluster. Genotype AP-17 admixed with UNK-NW accessions whereas AP3, AP4, AP11 and AP16 formed a separate group. Based on the grouping of UNK-NW and UNK-NE accessions with AG-S in the larger cluster we hypothesize these morphologically distinct genotypes growing near the single specimen of *A. pachyceras* in SANR area to be *A. gerrardii.* Pairwise genetic distance (Nie’s D) between the genotypes ranged from 6 (AP09-AP10) to 77 (AP07-AP12) (**Table S3**). We constructed a split decomposition tree on this genetic distance and observed AP01 and AP12 to be closely related. The grouping of other genotypes was in agreement with the PCoA analysis (**Figure 5B**).

Analysis of molecular variance (AMOVA) partitioned the genetic diversity within populations to be 75% whereas, among population diversity was 25% with a PhiPT (r) equalling to 0.249 (p<0.001). Total 999 permutation events were performed on 17 samples and 189 loci. Pairwise genetic distance within populations were 47 (AP-MT), 34.2 (UNK-NW), 25.4 (UNK-NE) and 30 (AG-S).

### DNA content and ploidy by flow cytometry

**Figure 6:**
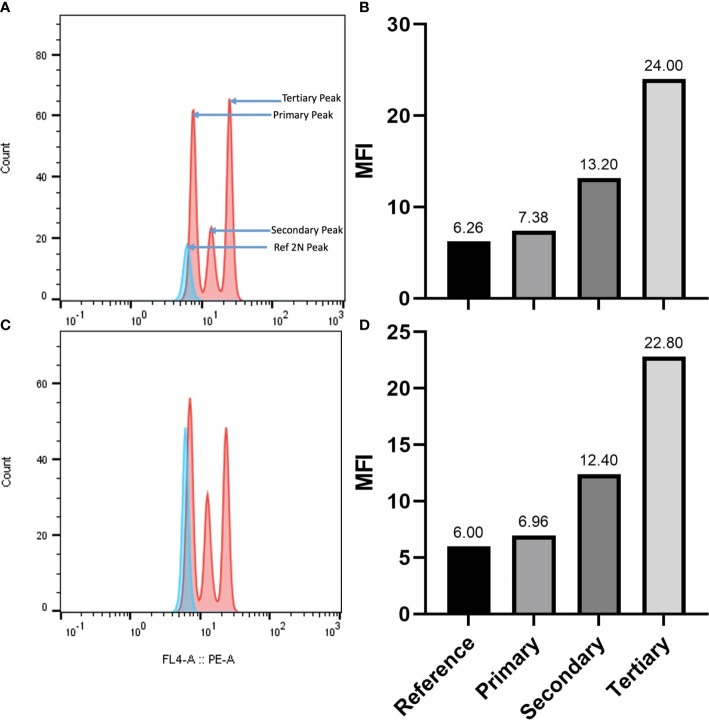
**(A)** Flow cytometric histograms for genome size estimation and ploidy assessment of *A. pachyceras.* The pink peaks correspond to the *A. pachyceras* nuclie whereas the blue peak represents the standard *Panicum miliaceum* (2C=2.09 pg; 2n=4x=36). The nuclei of reference and sample species were extracted and stained with propidium iodide and analyzed simultaneously. Relative fluorescence is plotted on the x-axis and the nuclei count are shown on the y-axis. **(B)** Bar plots of minimum fluorescence intensities of *A. pachyceras* and *P.miliaceum* nuclei. **(C)** Flow cytometric histograms for genome size estimation and ploidy assessment of *A. gerrardii.* **(D)** Bar plots of minimum fluorescence intensities of *A. gerrardii* and *P. miliaceum* nuclei.

The genome size of *A. pachyceras* was also estimated through flow cytometry with reference to the standard *P. milaceum* (**Table 3**). The 2C content of *A. pachyceras* was calculated as 2.46 pg DNA with a 1C value of 1.23 pg DNA. The holoploid genome size was estimated as 1204.9 Mb by applying a factor of 1 pg of DNA equivalent to 978 megabases. As compared to the standards’ 2n peak, *A. pachyceras* showed three distinct peaks suggestive of polyploidy in the collected specimen. The first peak or G1 peak is the diploid peak, whereas the second and the third peaks are most likely due to tetraploidy and hexaploidy. The median florescent intensity (MFI) of primary peak (G1 peak) had slightly higher value of 7.38 than the reference peak (6.26) whereas the secondary and tertiary peaks for *A. pachyceras* exhibited MFI equivalent to 13.20 (1.7 times higher) and 24 (3.2 times higher) respectively (**Figures 6A, B**). The difference in peak heights is attributed to different cells exhibiting varied ploidy levels in the same plant tissue. Based on the ploidy level analysis the 1Cx pg of DNA (monoploid DNA content) was estimated as 0.41 pg suggesting a monoploid genome size of *A. pachyceras* as 400.98 Mbp. Ploidy and genome size of *A. gerrardii* were also analyzed through flow cytometry. The 2C, 1C and 1Cx values were in parallel with *A. pachyceras* (**Figures 6C, D**). This species also possesses a hexaploid genome alike *A. pachyceras*.

**Table 3:** Genome size characteristics based on flow cytometry.

Species	2C-value (pg DNA)	1C-value (pg DNA)	1C-value (Mbp)	Ploidy level	1Cx -value (pg DNA)
*Acacia pachyceras*	2.46	1.23	1204.9	6	0.41
*Acacia gerrardii*	2.42	1.21	1185.5	6	0.40

2C and 1C pg DNA- mean holoploid genome size; 1pg=978 megabases; 1Cx value pg DNA= monoploid genome sizes (2C/ploidy).

## Discussion

Whole genome sequencing in several tree species has aided in understanding the evolutionary history, population genetics of rare and threatened species, molecular taxonomy, fragmentation, role of pollen dispersal, restoration management and adaptation to climate change (Byrne, 2018; Onley et al., 2021). Only few reports are available on these aspects of conservation management in *Acacia.* Where traditional genome analysis relies on relatively small amounts of deoxyribonucleic acid (DNA) of moderate purity, on the other hand, NGS technologies requires the input of several micrograms of high molecular weight DNA with a purity ranging between 1.8-2.0, free from polysaccharides and polyphenols (Healey et al., 2014). Several species of *Acacia* are woody trees that accumulate these contaminants in significant quantities limiting the study of their genomes (du Plessis et al., 1999; Byrne et al., 2001; Kumar et al., 2018; Maximo et al., 2020). In our previous, reports we came up with an optimized protocol to get desirable quality and quantity DNA for high throughput sequencing of *Acacia* (Habibi et al., 2022b). The present manuscript describes the applicability of our preceding research.

Genome survey sequencing provides preliminary understanding of the genomic characteristics of a species without prior knowledge before its large-scale sequencing can be performed. With a similar objective, high-quality libraries (Phred Score >30) of *A. pachyceras* were constructed and whole-genome sequencing was performed at a depth of 92x. The genome size of *A. pachyceras* was predicted as 720Mb which is quite comparable with *A. acuminata* (750Mb) and *A. melanoxylon* (750Mb) (Ferguson et al., 2021). On the contrary it was higher than the genome of *Vachellia collinsii* (462Mb) (Leichty and Poethig, 2019). The k-mer method has been used for predicting the genome size of non-model species (Liu et al., 2013; Lu et al., 2016) and was therefore, employed in the present study as well. Till date only two *Acacia* species from Australia have been sequenced at a whole genome level at a coverage of ~50X on an Oxford Nanopore MinION sequencer (Ferguson et al., 2021). At a similar coverage *V. collinsii* was sequenced on an Illumina HiSeq (Leichty and Poethig, 2019). Our results provide a valuable resource for high throughput sequencing studies in other species of *Acacia*. The chloroplast genome of *A ligulata* was predicted as 0.158Mb and that of *A. crassicarpa* as 0.176 Mb (Williams et al., 2015; Yue et al., 2021). DNA flow cytometry is another method for estimating the DNA quantity in cell nuclei and predict the genome size of an organism (Doležel and Bartoš, 2005). The flow cytometry anticipated the genome size of *A. pachyceras* as 1.2Gb. Differences in k-mer based and flow cytometric genome values were recorded in *Aspalathus linearis* (Mgwatyu et al., 2020) *Reseda pentagyna* (Al-Qurainy et al., 2021) and *Reseda lutea* (Al-Qurainy et al., 2021).

The GC content is an important feature of an organism’s genome. The identification of GC content and the factors behind its percentage distribution in the genome help elucidate the evolutionary status of a species. The GC content of 20 plant species was compared by Singh and his team (Singh et al., 2016). Their work demonstrated the GC content of grasses to be highest followed by non-grass monocots and dicots. The GC content of 35% in *Acacia* genome was close to *Populus trichocarpa* (33.71%), *Carica papaya* (34.91%) and *Vitis vinifera* (34.57%) (Singh et al., 2016).

Plant genomes are large in size, heterozygous, differ in ploidy levels, and highly repetitive making their assembly quite onerous (Michael and VanBuren, 2020). Presently, the *A. pachyceras* genome was assembled using MaSuRCA-4.0.3 that combines the benefits of the deBruijn graph and Overlap-layout-consensus. Moreover, the jellyfish mer-counter is also integrated within MaSuRCA. Similar algorithm was used to assemble the genome of *V. collinsii* (Leichty and Poethig, 2019). Unlike our results the assembly method of Canu v2.0 was used to assemble the genomes of *A. acuminata* and *A. melaxnoxylon.* However, the latter is specifically designed for high-noise single molecule genome sequenced on PacBio RSII or Oxford Nanopore MinION. Cross study comparisons in such cases are equivocal. We recorded an excessive repetitive rate of ≥ 50% and a heterozygosity ratio of 6.8% in the assembled genome of *Acacia.* Our future approach on sequencing the *Acacia* genome at single molecule level and physical mapping are likely to provide chromosome level assembly offering a better understanding of the genome complexity of the tree species under current investigation.

The flow cytometry analysis provided information on the DNA content in *A. pachyceras.* The 2C content of 2.46 pg of DNA observed in the present sample was close to the values obtained for *A. senegal* growing in Sind, Pakistan (2.89-2.99 pg of DNA) and Jodhpur, India (3.0 pg of DNA). However, two other accessions of Jodhpur, India showed a lower 2C content of 1.35 and 1.51 pg. The South African *A. senegal* depicted a 2C content ranging between 1.51 to 1.61 pg whereas the West African genotypes were in the range of 1.34 to 2.90 pg. Similar values were recorded for Central and East African populations (Odee et al., 2015). The 2C content of some other species of Leguminosae family were 4.42 pg DNA (*Galega oficinalis*), 1.60 pg DNA (*Lupinus polyphyllus*), 3.49 pg DNA (*Medicago sativa*), 1.09 pg DNA (*Trifolium hybridum*), 6.23 pg DNA (*Vicia grandiflora*) (Kubešová et al., 2010). All these species exhibited varied ploidy levels. The presence of multiple peaks in the k-mer frequency plot of *A. pachyceras* was suggestive of polyploidy in the species. The flow cytometric inferences also explained the possibility of hexaploidy in the only surviving specimen of *A. pachyceras.* Diploid, triploid, tetraploid, hexaploid and octaploid genomes were observed in *A. senegal* collected from sub-Saharan Africa, Pakistan, India and the Australian acacia, *A. dealbata* (Odee et al., 2015). Karyological investigations are warranted to derive confirmatory conclusions on the ploidy characterization of woody tree species of *A. pachyceras.*

SSRs, also known as microsatellites, are among the most useful and versatile genetic markers used in plant functional genomics. However, the identification of SSRs and their development using conventional techniques are arduous, expensive, and time-consuming (Taheri et al., 2018; Mathur et al., 2013; Habibi et al., 2020). High-throughput sequencing technologies have recently made it possible for researchers to identify thousands of microsatellites at a lesser cost with minimal efforts in a matter of few minutes as compared to the traditional BLAST search (Habibi et al., 2022a, c, Šarhanová et al., 2018; Taheri et al., 2018). We demonstrated the utility of genome sequencing to filter more than 900,000 SSR motifs. A total of 11,181 markers were successfully designed against these regions, a hundred of which amplified 186 genetic loci reasonable for genetic diversity analysis (Al Salameen et al., 2022; Al Salameen et al., 2020; Al Salameen et al., 2018a; Al Salameen et al., 2018). Our results of genetic diversity analysis pave a way for prospective research in conservation management of the rare and endangered *A. pachyceras*. SSR markers are co-dominant and exhibit cross transferability, hence can be applied for molecular research of other species of *Acacia*. This feature was also demonstrated in our investigation, as the markers successfully amplified the genetic loci of closely related *A. gerrardii* (Suleiman, 2017; Suleiman et al., 2018). Genome sequencing assisted in forest management and restoration of Hawaiian koa (*Acacia koa*) (Gugger et al., 2018).

## Data availability statement

The original contributions presented in the study are publicly available. This data can be found here: NCBI, PRJNA754103.

## Author contributions

NH: conceptualisation original draft preparation, reviewing and editing. FZ, AS, NR, NV and MR: methodology. NH and NV: software, data curation. NH: visualisation. NH and FS: validation. NV and VK: formal analysis. VK: investigation. FS: resources. FS: project administration. NH and FS: funding acquisition. All authors contributed to the article and approved the submitted version.
