# Supplementary material for: Genome survey and genetic characterization of Acacia pachyceras O. Schwartz
Source: Front Plant Sci. 2023 Feb 16;14:1062401. doi: 10.3389/fpls.2023.1062401 (PMC9979705; doi:10.3389/fpls.2023.1062401)
Supplement: Supplementary file 1 [file Table_1.docx]

**Supplementary Data**

**Table S1: Sampling location and morphological parameters of Acacia growing under field and green house condition**

| **Sample Code** | **Location** | **GPS Coordinates** | **Age** | **Morphology** | **Type** |
| --- | --- | --- | --- | --- | --- |
| AP1 | SANR | 29°34’909” N, 47°47’734” E | Mature | Small leaves, a smaller number of thorns | Wild |
| AP2 | KISR | 29°33’716” N, 47°90’602” E | 1 year | Small leaves, a smaller number of thorns | Nursery grown |
| AP3 | SANR | 29°34’909” N, 47°47’736” E | 4-5 years | Broad leaves, several long and thorny spines | Wild |
| AP4 | SANR | 29°34’909” N, 47°47’737” E | 4-5 years | Broad leaves, several long and thorny spines | Wild |
| AP5 | SANR | 29°34’909” N, 47°47’739” E | 4-5 years | Broad leaves, several long and thorny spines | Wild |
| AP6 | SANR | 29°34’909” N, 47°47’738” E | 4-5 years | Broad leaves, several long and thorny spines | Wild |
| AP7 | SANR | 29°34’909” N, 47°47’736” E | 4-5 years | Broad leaves, several long and thorny spines | Wild |
| AP8 | SANR | 29°34’909” N, 47°47’732” E | 4-5 years | Broad leaves, several long and thorny spines | Wild |
| AP9 | SANR | 29°34’909” N, 47°47’731” E | 4-5 years | Broad leaves, several long and thorny spines | Wild |
| AP10 | SANR | 29°34’909” N, 47°47’733” E | 4-5 years | Broad leaves, several long and thorny spines | Wild |
| AP11 | SANR | 29°34’909” N, 47°47’733” E | 4-5 years | Broad leaves, several long and thorny spines | Wild |
| AP12 | SANR | 29°34’909” N, 47°47’734” E | 4-5 years | Broad leaves, several long and thorny spines | Wild |
| AP13 | KISR | 29°33’716” N, 47°90’602” E | 2-6 months | Broad leaves, several long and thorny spines | Nursery grown |
| AP14 | KISR | 29°33’716” N, 47°90’602” E | 2-6 months | Broad leaves, several long and thorny spines | Nursery grown |
| AP15 | KISR | 29°33’716” N, 47°90’602” E | 2-6 months | Broad leaves, several long and thorny spines | Nursery grown |
| AP16 | KISR | 29°33’716” N, 47°90’602” E | 2-6 months | Broad leaves, several long and thorny spines | Nursery grown |
| AP17 | KISR | 29°33’716” N, 47°90’602” E | 2-6 months | Broad leaves, several long and thorny spines | Nursery grown |

**Table S2: Primers used for PCR amplification.**

| **S. No.** | **Left Primer** | | | **Right Primer** | | |
| --- | --- | --- | --- | --- | --- | --- |
|  | **Sequence** | **Tm** | **Start** | **Sequence** | **Tm** | **Start** |
| **1** | GGTAAGGCAATGCACAGAGG | 58.90 | 53 | TGATGTTGGACAAATGCATGGT | 59.10 | 295 |
| **2** | CAGCAGTGTAAAAGGCATGC | 58.01 | 322 | TGTGACCAGTTGCTCTCCAT | 58.94 | 529 |
| **3** | AGGCCTTGGTCATTTGGTTT | 57.90 | 259 | TCAATGGACTATCGCCGGAG | 59.33 | 484 |
| **4*** | AGGGTACATCATCGCTTGGT | 58.80 | 75 | AGTCACGAAAGCTGAGAAGGA | 59.04 | 291 |
| **5*** | TCAGATTCATTGGCGTTGCA | 58.47 | 110 | ACTAGTGTGAGCGGTTGAGA | 58.38 | 501 |
| **6** | TGCAATCCAAAGCTGTGACA | 58.31 | 302 | TCACGTGTTTAATCCTGCTTGT | 58.53 | 495 |
| **7** | CCATGCTCGTCGTTCACTTT | 58.85 | 311 | CGTTGTGTTCGCGAATGTTG | 58.97 | 554 |
| **8*** | TGGAGCATGGACATCAGAGG | 59.16 | 215 | GTCAAAAGACAATTGTAACCCGT | 57.82 | 379 |
| **9** | ACGATTTCAATGCTGTTGGTAGT | 58.93 | 453 | CATCATCACAACCTCGGCAT | 58.93 | 606 |
| **10*** | ACAGGAAAGAGGGAGGGAGA | 59.21 | 312 | GCTGGCGTTTATTTGGTGTG | 58.30 | 461 |
| **11** | TCAACCTCACGTCCAAGATCT | 58.75 | 18 | AGCCATTAAAAGCGTGCACT | 58.75 | 221 |
| **12*** | CCGCTGCCATGGAAGAATTT | 59.18 | 154 | GTGGTTCCTGCACTCCTCTC | 60.04 | 367 |
| **13** | TTTCCTTCGCCTTCTCCCAT | 59.00 | 231 | ACTGTCATCGCATGTCAAAATT | 57.23 | 437 |
| **14** | AGAAACTGACTCTTACTTCCCCT | 58.51 | 283 | TCGCATGACAAGAGAGGACT | 58.45 | 464 |
| **15** | TTGAATCCCTTCCCCATCGG | 59.45 | 449 | GGGTAGAGAAAGGTCCAGGT | 58.05 | 604 |
| **16*** | GAACAGAGAAGTTGCCGGTG | 59.13 | 502 | GCTTAGTGACCTCTGATTTCTCA | 57.92 | 678 |
| **17*** | CCTTCACAATAGGGCATCACT | 57.71 | 378 | TGAGAAGTTTGTTGGGGCCT | 59.44 | 627 |
| **18** | TGCATAAGACTGGGCATGGA | 59.08 | 4 | ACTTTGTTTTGGTTCACTTGACA | 57.54 | 274 |
| **19** | TATAGGGCAGTACACCGCAC | 59.25 | 316 | AAGACTTTTCCTCACTCTTGTCA | 57.64 | 472 |
| **20** | GCACAACCTCACAAACAAACC | 58.73 | 251 | AGCAGGACAGTGAGCATTTT | 57.72 | 590 |
| **21** | ACGCTTGTTTCACCAATTGC | 58.15 | 39 | ACGGGATGCAAGTAAGAATTCC | 58.72 | 190 |
| **22** | GCTACCCACCGTCTGATTCT | 59.18 | 391 | AGTGATGGTGACGGGTGATT | 59.02 | 622 |
| **23*** | ACCAGTTGATCCACCTGCAA | 59.52 | 507 | GAAGTGACAGCTGCCTTACG | 58.93 | 717 |
| **24** | CTTGTCAGGCTAAAATCCATGGA | 58.73 | 71 | CACAGAGAAGGAATGGCAGC | 58.91 | 294 |
| **25*** | AGCCAAATTCTTCCACTTGTCA | 58.43 | 126 | AACATCAAGGAGTGGCTCGA | 59.02 | 332 |
| **26** | TCACATTTATGGCACTCGACA | 57.67 | 609 | CAGAGAATGATAATGGCGGCA | 58.50 | 722 |
| **27** | TGCGCAGAGACAAGGTAGTT | 59.32 | 333 | ACCGTCAGTAAGTTCACCGT | 58.96 | 551 |
| **28*** | CTTTCCATGTAAACGGACCCA | 58.21 | 319 | ACCAGCGCCAATTACCAATT | 58.44 | 532 |
| **29*** | GCAACCTGAGATGAGAAGCG | 58.99 | 448 | GACGGTGATTTGGTCGAAGC | 59.56 | 608 |
| **30*** | CTTGAGGACATGGTGTTGGTC | 58.85 | 74 | ACCACTACAAATGCCACGTT | 58.02 | 272 |
| **31*** | GAAGAGGGTCGGATGTAGGA | 57.93 | 281 | GACTGGAGCCACAACAAAGT | 58.32 | 566 |
| **32** | TGCCAGTATCAAGCATGCTC | 58.33 | 267 | AGATGACGAGTGTGAAGGGA | 58.08 | 498 |
| **33*** | CGCGATTTCCAATGTACCGT | 59.00 | 123 | AGGTGGTGCGCATGTATGTA | 59.46 | 509 |
| **34*** | GCACCAACATGTAGTCGCAA | 59.13 | 355 | GAATGGGGAGGAGAAACGGA | 59.09 | 596 |
| **35** | ATGACCCTCCTCTACATCGT | 57.24 | 220 | TGTTACACGCACTTTCCAGG | 58.41 | 533 |
| **36*** | TGAACCGGCTTGCTTGATTT | 58.67 | 162 | TGGTTGGTAAAGGCTAGGAAC | 57.57 | 393 |
| **37*** | AGGAAAAGGGAAGGAGGCAA | 58.84 | 58 | GGCGTGAAGTAATGAGCGAG | 59.08 | 245 |
| **38** | AGAAGATAAGGAGAGATCCAGGT | 57.58 | 28 | GGGACGACAATCAGACAGGA | 59.10 | 194 |
| **39** | TCCTGTCTGATTGTCGTCCC | 59.10 | 175 | CACTGCAACGTCTTAGAGGC | 58.93 | 384 |
| **40** | TCACTCAATGGCCCAGAAAG | 57.79 | 446 | TGGGCTTTGGATTCGTTGAA | 58.01 | 675 |
| **41** | GCTTCCTCAAGAACAATCCCA | 58.20 | 224 | GATTAAGTCCGCTCGCTTGG | 59.07 | 450 |
| **42** | TGAGGAAGACTGCTACGGAA | 58.08 | 116 | ACCCAAATGAGCTGCTTCAA | 58.00 | 280 |
| **43** | ACACTGGAGATGCTGATTTGG | 58.28 | 83 | TGGGATGACTACCACTAAGACC | 58.63 | 267 |
| **44** | TTGGGCTTGGAGTCGGAG | 58.93 | 19 | TACAGGCTTCTTTTGCACGC | 59.41 | 171 |
| **45** | ACTCCAATGCTAGTGACCCA | 58.34 | 329 | TGGAGTTTAAAAGGTCAAAGCAA | 57.00 | 605 |
| **46** | TCACACGACTTACCCATTAACC | 58.07 | 54 | TCCATATTCTCGTCTGCATTGAC | 58.63 | 232 |
| **47** | GGCTCCCATCCGATGAATTC | 58.47 | 20 | ATTGGCCACTCGAGAAAACC | 58.47 | 189 |
| **48** | TCTGTTGGAGATGGCGTCA | 58.64 | 157 | TAGCATCAGGGTATGGGAGC | 58.65 | 389 |
| **49** | ACACAGGTCCATCAATTAACATG | 57.10 | 19 | GCAAGATGTGATGTGCGC | 57.63 | 268 |
| **50** | TGAATTTCAAAGAAGTGGCCAAA | 57.65 | 40 | AAGGACACGAGGTAGTAGTACT | 57.43 | 216 |
| **51*** | TGGCTCTCACGGTTCAATCA | 59.32 | 202 | TGTTGACGGTGAAGGAAGGA | 58.88 | 353 |
| **52** | TGGGCTACAACAGTCGAAGA | 58.67 | 55 | TTTGGTGCATGGGTTACAGG | 58.37 | 314 |
| **53*** | CGACGACAACAAAGCCAACT | 59.34 | 11 | AGGGATTTATGCTGTCACTTTGT | 58.33 | 181 |
| **54** | GGTGGGATGGGATTGCCTTA | 59.44 | 569 | TGCTTGAATGTGTACCAGCT | 57.43 | 740 |
| **55** | CAGCTCTCTCTGCCTCAAGT | 59.10 | 646 | GGGATGAGTTGCTGCACTTT | 58.75 | 885 |
| **56** | ATCGTTGGTTTGCTGCAGTT | 58.97 | 48 | ACTGAACCTTTGCTGGCATG | 59.04 | 266 |
| **57** | CATGCCAGCAAAGGTTCAGT | 59.04 | 247 | GAAGGCAGCAGTGTTGATCC | 59.19 | 471 |
| **58** | TTGCTCCTTAAAATGCGTACAG | 57.32 | 177 | AAGGAGCAGTGATCGATGGG | 59.53 | 403 |
| **59** | TCATGGTTCTGAGTCCGTCA | 58.37 | 26 | ACGTTCCCAGAGAGATTTCCT | 58.46 | 216 |
| **60** | GGGCCTCCAAAACATGAAAGA | 58.75 | 288 | TGAATCCTGTCAAGCATGCT | 57.50 | 486 |
| **61*** | ACCCATCCTTGTTGCTGAGA | 58.93 | 262 | AGCACAAATCACATTCAAAACCA | 58.93 | 506 |
| **62** | CCCACTCTCTCCCCATGTTT | 59.00 | 356 | CCTTCTTACCAGCCTCACCA | 59.02 | 509 |
| **63** | CTTTCAACGGTGGCTTCTACA | 58.51 | 4 | CTTTCAACGGTGGCTTCTACA | 57.63 | 210 |
| **64*** | AAACCGAATAGTGGCTGCAC | 58.53 | 22 | AGTTTTCCCCGGCTCACTAA | 58.94 | 197 |
| **65** | TGAATGTGTGCGTAAAAGGGT | 58.42 | 51 | GGGTAGCTACACACACATGG | 57.98 | 271 |
| **66** | CGAAATCGCGTTGGACATTT | 57.76 | 235 | CTCACTTGTATTGGCTCGCC | 58.99 | 448 |
| **67** | GAAGGCTCTGTTTCGTGCAA | 59.06 | 7 | TGTGCCCATGACAGAACATTG | 59.11 | 187 |
| **68** | TCAGGCCCAAGATCTCCATT | 58.40 | 711 | ACAAGAGATTAAGATGGCGAGAG | 58.06 | 994 |
| **69*** | CAACGAAAGGCTCCTGGAAC | 59.12 | 154 | TCCTTATGCTCCTCCACGTT | 58.43 | 304 |
| **70*** | GGCAATGGGGAAGGAAAAGG | 59.10 | 213 | TGGAGGAAGGAGAAGATGACG | 58.90 | 442 |
| **71*** | TGGACGAGAGAATAACACGGA | 58.56 | 409 | TCCCTATTTTGTGAGTGCCC | 57.49 | 596 |
| **72*** | CTCCCCTTCAGGCGTTCTAA | 59.1 | 274 | GCTCAGGAGGCTAATGGGAA | 59.16 | 482 |
| **73** | GAGTATACCAAGGACCAAGTTCA | 57.58 | 29 | TGGGCGAGGTTCTTAGTAGG | 58.52 | 232 |
| **74** | TGAGTCTTGAAGGCCTTTTGTC | 58.79 | 230 | ATGACAGACCTAAGGCCTCT | 57.44 | 526 |
| **75** | TCCATTAAGTCCTAGAACGCAA | 57.19 | 365 | ACCCTGATCGTTAATTCTCTGC | 58.2 | 600 |
| **76** | AAAGGGGTGCGGTCTAGAAA | 58.94 | 356 | ACAGGGCTTAGAAAGAACACTT | 57.56 | 577 |
| **77** | CCCGAGGTCCCAAATTCAAA | 58.08 | 386 | TCAAGAGAGGAGTTGCAGCC | 59.68 | 563 |
| **78** | TGTGGAAAGTTGTTGAGGTGT | 57.92 | 283 | TACCCAGAGAGCCACTTGTC | 58.73 | 531 |
| **79** | TTCGTGTGTGTGTGTGTGTG | 59.21 | 326 | TACCCAGAGAGCCACTTGTC | 58.73 | 531 |
| **80** | AATGCTTCCATGTGCTTGCT | 58.74 | 86 | TGAGTGGTGGAAGAGACGTC | 59.04 | 240 |
| **81** | CACTCATGCCTAGGTTGGAA | 57.21 | 283 | TGGCCCTTTGAAAATCTGCA | 58.29 | 450 |
| **82** | GCTGTGACCATTTCTGCTCT | 58.18 | 122 | GTTGACATACGCGATTCCTGA | 58.46 | 336 |
| **83** | ACAGGGATTCAAGTTCTCGGT | 59.02 | 283 | CGCAAATGAAATAACAAGCTGGT | 58.76 | 501 |
| **84** | TGTATGCTTGTCTTCCTCATGG | 58.12 | 431 | GGGTTGGGATTGTTAAATGAGC | 57.88 | 660 |
| **85** | TAGACCCAAAACGGCATCCA | 59.31 | 78 | GGTCTGGGCTGAAAACGTTT | 58.97 | 246 |
| **86** | TGAGTGATTTTGCCAAACATGG | 58.01 | 263 | CGATCGGACTAGAATCGGGT | 58.76 | 469 |
| **87** | ACGTTGAACTAAACCTTTTCCCT | 58.47 | 54 | GCTTAAAGGGTGCCGTACAA | 58.47 | 239 |
| **88*** | ACCTTGCTGCAAATTTCGGT | 58.96 | 96 | GATCAAGTTAGTGCGCTGGT | 58.27 | 319 |
| **89*** | TGTGTGTGTGTGTGTGTGTT | 58.4 | 215 | AGAAACCAGCAGCCAATGTC | 58.75 | 464 |
| **90** | TCTTCTCCCTCTTGCTGCAA | 58.94 | 137 | GAGGCATCTTCGTTCTCAGC | 58.71 | 297 |
| **91** | AGCAGGCTAACAAACCATAAACA | 58.85 | 115 | TGTAAACAAGTCAAACGGCCT | 58.35 | 318 |
| **92** | GCCCATTTATCCCTCCCTGA | 58.85 | 177 | GAAGCATTAACTGTGTGAAGACA | 57.33 | 420 |
| **93*** | CGCAAAGAGAGAGAGAGGGA | 58.53 | 45 | GGCCGACCCGTTTAGAGAC | 60.15 | 224 |
| **94*** | GGCCCACTCATTTCACTTGG | 59.11 | 59 | AGGGATGCCGATGAAGAACA | 59.09 | 234 |
| **95** | TGACACATGATGCATTTGATGAT | 57.15 | 135 | CGCAACCGTTAAGTCTGTAGT | 58.33 | 301 |
| **96** | CCGTTCCAACCCTTCATTGT | 58.38 | 297 | TTTTGGAGCTTGTAACGGGC | 59.04 | 499 |
| **97** | GGTCGATGTGGTGCTGTTAC | 58.92 | 348 | TTGGCCATTCTTCAAACCGT | 58.30 | 578 |
| **98** | TCAGCAGTAGTGGATGTGGG | 59.10 | 91 | CCTACGCCTTTGCCTTCATC | 58.98 | 299 |
| **99** | TGATGATGAAAGGGGCCTCT | 58.40 | 811 | GGGGAAAGAGGTGGTTGGAT | 59.30 | 1003 |
| **100** | TGACATACGTTGGGAGGGTT | 58.65 | 294 | CCCTTAGCCACTTTTCACCT | 57.41 | 487 |
| **101** | ACTTTCTTCATGGGTGGTCC | 57.41 | 905 | ACCTTTTCTCCCCTTCGATACT | 58.55 | 1127 |
| **102** | TCATGAAACACACACACCAGT | 58.00 | 97 | TCGTTTACAACTTGTGCGCT | 58.71 | 290 |
| **103** | AGATAGGTTCAATGTTGCCCC | 57.98 | 355 | CCACCGAAGACATGAAACGA | 58.21 | 505 |
| **104** | TGTGGAATGGGTCTTAATCTGAG | 57.90 | 175 | GCAAGGACCCCATTTTGAAGT | 59.03 | 353 |
| **105** | TGGCCTTTATTTCTGTTGACGA | 58.25 | 8 | TTCTAAAACGACCGCAGCAC | 59.14 | 208 |

* Primers yielding polymorphic bands in the current analysis

**Table S3 Pairwise genetic distance between 17 genotypes of *Acacia* as revealed by GenAlEx 6.5 software**
